# Alcohol ban during the COVID-19 pandemic lockdown: Lessons for preventing foetal alcohol spectrum disorder in South Africa

**DOI:** 10.4102/phcfm.v14i1.3686

**Published:** 2022-12-20

**Authors:** Babatope O. Adebiyi, Ferdinand C. Mukumbang

**Affiliations:** 1 Centre for Interdisciplinary Studies of Children, Families and Society, Faculty of Community and Health Sciences, University of the Western Cape, Cape Town, South Africa; 2 School of Public Health, Faculty of Community and Health Sciences, University of the Western Cape, Cape Town, South Africa

**Keywords:** Foetal alcohol spectrum disorder (FASD), policy, prevention, South Africa, alcohol ban, national lockdown, COVID-19, alcohol consumption, pregnancy

## Abstract

During the two national lockdowns implemented in South Africa to curb the spread of the coronavirus disease 2019 (COVID-19) pandemic, the sale and consumption of alcoholic beverages were prohibited. There is observational evidence from the literature suggesting a drastic reduction in the emergency and trauma unit admissions in many South African hospitals and clinics with alcohol-related restrictions. This article explores the potential benefits of the restrictions placed on the sale and consumption of alcohol during the COVID-19 pandemic on preventing foetal alcohol spectrum disorder (FASD) in South Africa. Following the potential benefits of the alcohol bans, we recommended that the current South African national liquor policy and the 2012 South African government-drafted Bill for Control of Marketing of Alcoholic Beverages should be fully implemented and enforced. Furthermore, the ‘best buys’ by the World Health Organization (WHO) should be adapted (based on local evidence) and executed. Implementing the abovementioned policies can reduce alcohol abuse by limiting and regulating the manufacturing, distribution, advertising, sponsorship, promotion, physical availability and hours of sale of alcoholic beverages in South Africa.

**Contribution:**

This article shows that alcohol bans during the coronavirus disease 2019 (COVID-19) lockdown reduced the short-term effects of alcohol. We believe that this could be a game-changer for the prevention of FASD in South Africa and positively impact the incidence and prevalence of FASD. This piece provides evidence that policymakers, health practitioners and academics can use to continue advocating for stricter alcohol control measures in South Africa.

## Introduction

On 11 March 2020, the World Health Organization (WHO) labelled the coronavirus disease of 2019 (COVID-19) a pandemic. National governments and the WHO introduced different measures to contain the COVID-19 pandemic. These measures included social or physical distancing, lockdown, testing, mask-wearing, isolation, quarantine, hand-washing, restrictions on gatherings and movements, closure of schools and the prohibition of alcohol sales.

Among the measures listed above, the alcohol sales and consumption ban was particularly questionable. The justification provided by the government was partly related to the role of alcohol in undermining social distancing and compromising immune response to COVID-19 infections.^1^ The government’s decision to continue the ban on alcohol during that period was strengthened by alcohol’s reported contribution to the high levels of interpersonal violence and injuries. Because alcohol consumption is strongly associated with violence-related trauma admissions in South African health care institutions, the ban was envisaged to free up health services to accommodate those infected with COVID-19.^2^ In addition, with men confined to their homes during the lockdowns, there were concerns about domestic violence associated with alcohol consumption^1^.

The South African government has struggled over the years to balance the short- and long-term consequences of alcohol against its economic benefit – income and employment.^3^ Therefore, the alcohol industry has continued to use the guise of economic benefits to lobby the government during decision-making. In South Africa, the rate of alcohol consumption is one of the highest among those who consume it worldwide. Sixty-five per cent of people between the ages of 15 and 19 who consumed alcohol reported binge drinking in 2016.^4^ Alcohol consumption during pregnancy is reported to range from 2.5% to 45%.^5^ Pregnant women continue to consume alcohol, because they either do not know that they are pregnant or use alcohol to cope with stress and other common mental health illnesses, such as depression and anxiety.^6^

Alcohol use disorder has been linked to many societal problems, such as violence, child abuse, injuries, risky sexual behaviour and foetal alcohol spectrum disorder (FASD).^4^ Foetal alcohol spectrum disorder is the leading cause of preventable developmental disabilities in the world. Foetal alcohol spectrum disorder refers to the range of effects that can occur because of prenatal alcohol exposure. The prevalence of FASD varies from country to country, with the global prevalence estimated to be 8 per 1000 children and youth in 2017. In South Africa, the national prevalence of FASD ranges from 29 to 290 per 1000 live births – the highest recorded prevalence of FASD globally.^7^

Foetal alcohol spectrum disorder may result in primary and secondary disabilities for individuals prenatally exposed to alcohol. Primary disabilities (those disabilities that the child is born with) may include abnormal facial features, learning disabilities, attention difficulties, poor memory, poor reasoning and judgement skills, hyperactive behaviour and poor coordination. Without appropriate interventions to mitigate the primary disabilities of FASD, secondary disabilities may develop. Foetal alcohol spectrum disorder–related secondary disabilities may include mental health problems, disrupted school experience, trouble with the law, confinement, inappropriate sexual behaviour and alcohol or drug problems.^8^

In this commentary, we discuss the possible relationship between the alcohol ban during the lockdown period and the short-term health risks of alcohol (motor vehicle crashes, falls, drowning, firearms, assaults, violence and burns). Based on the relationship, we hypothesise a potentially long-term benefit for reducing the incidence of FASD. We argue that observed reductions in the short-term health risks of alcohol during the national lockdowns indicate long-term health benefits, such as reduced incidence of FASD.

## The central argument

Following the ban on the sale and consumption of alcohol, there were reductions in alcohol-related health risks, particularly injuries (motor vehicle crashes, falls, drowning, firearms, assaults, violence and burns) and alcohol poisoning.^2,9,10,11,12,13^ The Sentinel Trauma Report clearly shows that the lockdown regulations and alcohol ban effectively reduced the number of trauma presentations by 40% – 50%.^11^ After lifting the alcohol ban, the absolute number of trauma cases requiring admission in the five hospitals (in the Western Cape) sampled over one month (June 2020) increased by 54.2% and the number of trauma deaths in the Eastern Cape province of South Africa increased by 307%.

Another study demonstrated lower trauma admission and operation rates during alcohol prohibition compared to nonprohibition during the COVID-19 pandemic over one year in a South African regional hospital (01 January to 28 December 2020).^12^ Whenever a complete ban was implemented, there was a substantial drop in trauma volume, which was lost by lifting the alcohol ban. For example, there was a 59% – 69% decrease in trauma volume between pre-COVID-19 and the first complete ban period (27 March – 31 May 2020). When alcohol sales were also partially reinstated, trauma volume significantly increased (83% – 90%), and the introduction of a second alcohol ban saw a 39% – 46% decrease in trauma volume. Trauma volume increased by 163% – 250% when alcohol sales were partially allowed again in the second half of 2020.^12^

The reductions observed in the above study were supported by a study conducted to show the impacts of alcohol bans and curfews on unnatural deaths from 01 December 2019 to 01 April 2021.^9^ The study reported a significant association between weekly unnatural deaths and complete restrictions on the sale of alcohol. Furthermore, the result of the study provided convincing evidence that the restriction on the sale of alcohol and not curfew is associated with the reduction in unnatural deaths observed during the COVID-19 pandemic in South Africa.^9^

The evidence presented above undoubtedly shows the impact of the lockdown alcohol ban on short-term alcohol-related health risks because of reduced availability and consumption. The relationship between the short- and long-term health risks has a common underlying factor – a decrease in availability and consumption – in reducing the health risks. Although the impact of the alcohol ban on FASD incidence was not explicitly, empirically assessed (this is because it cannot be done immediately), we submit that reduced availability and consumption of alcohol can decrease the incidence of FASD. Research evidence shows that reduced availability and consumption of alcohol will facilitate both short- and long-term health risks, including FASD.^14^

## Discussion

The ban on alcoholic beverages was, understandably, not well-received by the general population. However, this ban was a gesture appreciated by many stakeholders except, of course, the alcohol industry. Many experts, especially health and alcohol researchers and nonprofit organisations, considered the ban to be experimenting with measures to address alcohol-related issues in South Africa.^1^ They argue that some measures introduced during the lockdown can be implemented post-COVID-19 to reduce alcohol harm and, consequently, the incidence and prevalence of FASD.

The alcohol sector accounted for 3.4% of South Africa’s nominal gross domestic product (GDP) in 2019. Consequently, establishments involved in producing, distributing and selling alcoholic beverages cite the loss of jobs for individuals and income for the governments and increased illegal trading and home brewing^15^ to discourage alcohol bans. Nevertheless, the negative impacts of alcohol-associated economic, social and health costs are estimated to be 10% – 12% of South Africa’s GDP.^16^ According to the Foundation for Alcohol-Related Research, the health, financial and social implications of FASD are also enormous on individuals, families, society and the government. These financial consequences are estimated at R42 billion annually in financial terms. Looking at the costs associated with the negative impacts of alcohol, it could be argued that the negative consequences outweigh the positive effects.

There is also an argument that people with alcohol use disorders may suffer withdrawal syndrome from the sudden loss of access to alcohol during the lockdown.^1^ Such withdrawal syndromes can be addressed by providing accessible and affordable treatment for people with alcohol use disorder – one of the ‘best buying’ strategies proposed by the WHO.^14^

In this commentary, we argue that the ban on alcohol sales and consumption during the COVID-19 lockdown periods has potentially reduced the incidence and prevalence of FASD, considering the impacts that we have observed in other short-term health risks of alcohol.^14^ We believe that the lockdown has provided the South African government with opportunities to address the gaps identified in an FASD policy guideline and to implement existing policy recommendations for FASD in South Africa.^17^ Human rights activists have reported that the ban on alcohol sale and consumption does not violate human rights as long as it is done to protect children.^4^ While a total ban on alcohol sales and consumption is not sustainable, we believe that stricter alcohol control measures based on local evidence (such as an increased price of alcohol and excise duties) can be implemented in South Africa.

## Recommendation

The prevention and treatment of harmful alcohol use form part of the targets of Goal 3 (health and well-being – strengthen the prevention and treatment of substance abuse, including narcotic drug abuse and harmful use of alcohol) of the Sustainable Development Goals. Therefore, efforts should be made to regulate the use of alcohol in South Africa to achieve this target. Alcohol researchers, nonprofit organisations and stakeholders are calling for stricter alcohol control measures (reduction of trading hours, not a total ban) to reduce both the short- and long-term health risks of alcohol in South Africa. We therefore echo this position, as the COVID-19 pandemic has given us opportunities to test stricter alcohol control measures.

We suggest that the South African government implement and enforce the existing liquor laws. The lack of enforcement of liquor laws is one of the significant problems in the fight against harmful alcohol use. For example, the *Liquor Amendment Bill*, first introduced in 2016, proposes several wide-reaching changes, including: (1) increasing the drinking age to 21 years; (2) introducing a 100-m radius limitation of trade around educational and religious institutions; (3) banning of any alcohol sales and advertising on social and small media; and (4) the introduction of new liability clause for alcohol-sellers.

The 2012 South African government–drafted *Bill for the Control of Marketing of Alcoholic Beverages* should also be fully implemented and enforced. The bill is designed to contribute to the reduction of alcohol-related harm and the protection of public health and community well-being by limiting the exposure of the public to alcohol marketing by: (1) restricting the advertisement of alcoholic beverages; (2) prohibiting any sponsorship associated with alcoholic beverages (excluding donations); and (3) prohibiting any promotion of alcoholic beverages.

The South African government should also adopt (based on local evidence) evidence-based recommendations of the ‘best buys’ strategies proposed by the WHO in 2017. These include: (1) by regulating the marketing of alcoholic beverages (in particular to younger people); (2) by regulating and restricting the availability of alcohol; (3) by enacting appropriate drink-driving policies; (4) by reducing demand through taxation and pricing mechanisms; (5) by raising awareness of public health problems caused by harmful use of alcohol and ensuring support for effective alcohol policies; (6) by providing accessible and affordable treatment for people with alcohol-use disorders; and (7) by implementing screening and brief interventions programmes for hazardous and harmful drinking in health services.

## Conclusions

The national lockdown implemented by the South African government to curb the spread of COVID-19 provided an opportunity to implement various measures to reduce the availability and consumption of alcohol. Available evidence shows that these measures reduced the short-term negative impacts of alcohol. We believe that this could positively impact the incidence and prevalence of FASD in South Africa because of the relationship between the short- and long-term effects. Therefore, we call on advocacy groups to continue advocating for implementing some alcohol-related restrictions implemented during the lockdown to achieve a sustainable reduction in the incidence and prevalence of FASD. We also call on the government to implement and enforce the 2012 South African government–drafted *Bill for Control of Marketing of Alcoholic Beverages*.
